# Extended Follow-Up of Chronic Immune-Related Adverse Events Following Adjuvant Anti–PD-1 Therapy for High-Risk Resected Melanoma

**DOI:** 10.1001/jamanetworkopen.2023.27145

**Published:** 2023-08-03

**Authors:** Rachel S. Goodman, Aleigha Lawless, Rachel Woodford, Faisal Fa’ak, Asha Tipirneni, J. Randall Patrinely, Hui Ling Yeoh, Suthee Rapisuwon, Andrew Haydon, Iman Osman, Janice M. Mehnert, Georgina V. Long, Ryan J. Sullivan, Matteo S. Carlino, Alexander M. Menzies, Douglas B. Johnson

**Affiliations:** 1 Vanderbilt University School of Medicine, Nashville, Tennessee; 2 Massachusetts General Hospital Cancer Center, Harvard Medical School, Boston; 3 Department of Medical Oncology, Melanoma Institute of Australia, Sydney, New South Wales, Australia; 4 Department of Hematology and Medical Oncology, Perlmutter Cancer Center, NYU Langone Health, New York University School of Medicine, New York; 5 Department of Oncology, Lombardi Comprehensive Cancer Center, Georgetown University Medical Center, Washington, DC; 6 Department of Dermatology, Vanderbilt University Medical Center, Nashville, Tennessee; 7 Department of Medical Oncology, Alfred Health, Melbourne, Victoria, Australia; 8 Division of Hematology and Medical Oncology, Perlmutter Cancer Center, NYU Langone Health, New York University School of Medicine, New York; 9 Department of Medical Oncology, Melanoma Institute of Australia, The University of Sydney, Sydney, New South Wales, Australia; 10 Mater and Royal North Shore Hospitals, Sydney, New South Wales, Australia; 11 Division of Hematology and Medical Oncology, Massachusetts General Hospital Cancer Center, Harvard Medical School, Boston; 12 Department of Medical Oncology, Blacktown and Westmead Hospitals, Melanoma Institute of Australia; 13 The University of Sydney, Sydney, New South Wales, Australia; 14 Department of Medical Oncology, Melanoma Institute of Australia, The University of Sydney, Sydney, New South Wales, Australia, Mater and Royal North Shore Hospitals, Sydney, New South Wales, Australia; 15 Department of Hematology and Oncology, Vanderbilt University Medical Center, Nashville, Tennessee

## Abstract

**Importance:**

Anti–programmable cell death-1 (anti–PD-1) improves relapse-free survival when used as adjuvant therapy for high-risk resected melanoma. However, it can lead to immune-related adverse events (irAEs), which become chronic in approximately 40% of patients with high-risk melanoma treated with adjuvant anti–PD-1.

**Objective:**

To determine the incidence, characteristics, and long-term outcomes of chronic irAEs from adjuvant anti–PD-1 therapy.

**Design, Setting, and Participants:**

This retrospective multicenter cohort study analyzed patients treated with adjuvant anti–PD-1 therapy for advanced and metastatic melanoma between 2015 and 2022 from 6 institutions in the US and Australia with at least 18 months of evaluable follow-up after treatment cessation (range, 18.2 to 70.4 months).

**Main Outcomes and Measures:**

Incidence, spectrum, and ultimate resolution vs persistence of chronic irAEs (defined as those persisting at least 3 months after therapy cessation). Descriptive statistics were used to analyze categorical and continuous variables. Kaplan-Meier curves assessed survival, and Wilson score intervals were used to calculate CIs for proportions.

**Results:**

Among 318 patients, 190 (59.7%) were male (median [IQR] age, 61 [52.3-72.0] years), 270 (84.9%) had a cutaneous primary, and 237 (74.5%) were stage IIIB or IIIC at presentation. Additionally, 226 patients (63.7%) developed acute irAEs arising during treatment, including 44 (13.8%) with grade 3 to 5 irAEs. Chronic irAEs, persisting at least 3 months after therapy cessation, developed in 147 patients (46.2%; 95% CI, 0.41-0.52), of which 74 (50.3%) were grade 2 or more, 6 (4.1%) were grade 3 to 5, and 100 (68.0%) were symptomatic. With long-term follow-up (median [IQR], 1057 [915-1321] days), 54 patients (36.7%) experienced resolution of chronic irAEs (median [IQR] time to resolution of 19.7 [14.4-31.5] months from anti–PD-1 start and 11.2 [8.1-20.7] months from anti–PD-1 cessation). Among patients with persistent irAEs present at last follow-up (93 [29.2%] of original cohort; 95% CI, 0.25-0.34); 55 (59.1%) were grade 2 or more; 41 (44.1%) were symptomatic; 24 (25.8%) were using therapeutic systemic steroids (16 [67%] of whom were on replacement steroids for hypophysitis (8 [50.0%]) and adrenal insufficiency (8 [50.0%]), and 42 (45.2%) were using other management. Among the 54 patients, the most common persistent chronic irAEs were hypothyroid (38 [70.4%]), arthritis (18 [33.3%]), dermatitis (9 [16.7%]), and adrenal insufficiency (8 [14.8%]). Furthermore, 54 [17.0%] patients experienced persistent endocrinopathies, 48 (15.1%) experienced nonendocrinopathies, and 9 (2.8%) experienced both. Of 37 patients with chronic irAEs who received additional immunotherapy, 25 (67.6%) experienced no effect on chronic irAEs whereas 12 (32.4%) experienced a flare in their chronic toxicity. Twenty patients (54.1%) experienced a distinct irAE.

**Conclusions and Relevance:**

In this cohort study of 318 patients who received adjuvant anti–PD-1, chronic irAEs were common, affected diverse organ systems, and often persisted with long-term follow-up requiring steroids and additional management. These findings highlight the likelihood of persistent toxic effects when considering adjuvant therapies and need for long-term monitoring and management.

## Key Points

**Question:**

What are the incidence and spectrum of long-term outcomes of chronic immune-related adverse events (irAEs) from adjuvant anti–programmable cell death-1 (anti–PD-1) therapy?

**Findings:**

In this cohort study of 318 patients treated with adjuvant anti–PD-1 therapy for advanced and metastatic melanoma, 63.3% of patients with chronic irAEs (29.2% of all patients with adjuvant PD-1 therapy) experienced persistent irAEs with prolonged follow up.

**Meaning:**

These findings suggest that chronic irAEs are common and often persistent, emphasizing the importance of careful risk-benefit analysis and prolonged monitoring and management when considering adjuvant anti–PD-1 therapy.

## Teaser

This cohort study examined the incidence, characteristics, and long-term outcomes of chronic immune-related adverse events from adjuvant anti–programmable cell death-1 for advanced and metastatic melanoma at 6 institutions in the US and Australia.

## Introduction

Immune checkpoint inhibitors (ICIs) have become the preferred first-line treatment for advanced melanoma and produce durable antitumor responses in approximately 50% of patients.^1,2^ ICIs targeting programmed death-1/ligand-1 (PD-1/PD-L1) prolong recurrence-free survival (RFS) when used as adjuvant therapy.^3,4,5^ Anti–programmed cell death-1 (anti–PD-1) also causes widespread T-cell activation and results in autoimmune side effects involving multiple organs, termed immune-related adverse events (irAEs). While most severe irAEs are acute and resolve with glucocorticoids, we recently reported that up to 43% of irAEs persist for at least 12 weeks following therapy cessation in patients with melanoma treated with adjuvant anti–PD-1.^6,7^

Given the expanding use of anti–PD-1 across various tumor types, it is imperative to further assess its long-term effects, which have not been well defined. To address this gap, we conducted a large, multicenter cohort study with extended follow-up in patients with chronic irAEs from adjuvant anti–PD-1 therapy. This study aimed to determine the incidence, characteristics, and long-term outcomes, including resolution vs persistence, of chronic irAEs from adjuvant anti–PD-1.

## Methods

This cohort study followed the Strengthening the Reporting of Observational Studies in Epidemiology (STROBE) reporting guideline. Institional review board or ethics committee approval was obtained from each institution, and informed consent was waived because patient data were deidentified.

Retrospective deidentified data were collected from 6 participating academic medical centers in the US and Australia. Patients who received at least 1 dose of adjuvant anti–PD-1 were included. Those without evaluable follow-up for at least 18 months following treatment cessation were excluded. Patient demographics (melanoma subtype, comorbidities, genetic mutation status, and stage), treatment details (dose, frequency, duration, and reason for discontinuation), and disease recurrence details were collected from the electronic medical record from each institution. Type, grade, duration, treatment, and resolution details of acute, delayed, and chronic irAEs were noted.^8^ Acute irAEs were defined as those arising during treatment, delayed irAEs as those arising within 6 months after treatment cessation, and chronic irAEs as those extending at least 3 months after treatment cessation (Figure). In total, 318 patients were included. Of these, 304 patients were included with extended follow-up from our prior publication,^7^ and 14 were patients who were not included in the original publication but met the inclusion criteria from this study. Patients from the original cohort without a minimum evaluable follow-up duration of 1.5 years after treatment cessation were excluded.

**Figure.**
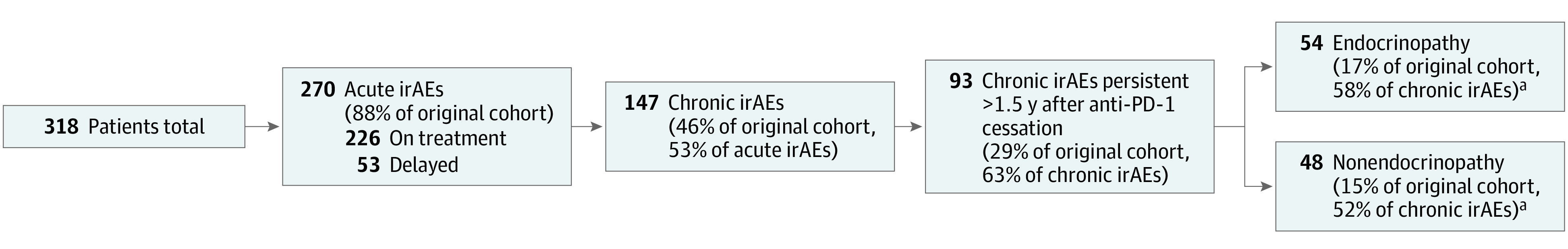
Acute, Chronic, and Persistent Immune-Related Adverse Events (irAEs) From Adjuvant Anti–PD-1 Therapy in Patients With High-Risk Resected Melanoma ^a^Nine patients experienced persistence of both endocrine and nonendocrine chronic irAEs at the time of last follow-up.

### Statistical Analysis

Descriptive statistics were used to analyze categorical and continuous variables. The IQR was calculated by subtracting the first quartile, the RFS or OS value below which 25% of the data fell, from the third quartile, the RFS or OS value above which 25% of the data fell. The start time for RFS and OS was the date of anti–PD-1 initiation. Kaplan-Meier curves assessed survival using GraphPad version 9.5.1 (GraphPad Prism). Wilson score intervals were used to calculate CIs for proportions.

## Results

Of the 318 patients included in this study, the median (IQR) age was 64 (52.3-72.0) years and 190 (59.7%) were male (eTable 1 in Supplement 1). Cutaneous melanoma was the primary in 270 patients (84.9%), 171 (53.8%) had BRAF/NRAS wild-type variants, 113 (35.5%) had resected stage IIIB, and 124 (39.0%) had resected stage IIIC melanomas (AJCC Cancer Staging Manual, version 8). Nivolumab (255 [80.2%]) and pembrolizumab (63 [19.8%]) were given with a median (IQR) treatment duration of 315 (170-351) days. There were 170 patients (53.5%) who completed 12 months of therapy. Treatment was discontinued for progression for 56 patients (17.6%), toxicity for 76 patients (23.9%), or other reasons, such as patient preference or comorbidities, for 16 patients (5.0%). Additionally, 193 patients (60.7%) had not experienced a recurrence at last follow-up, 56 (17.6%) experienced regional-only recurrences, and 69 (21.7%) experienced metastatic recurrences; 284 patients (89.3%) were alive at a median (IQR) follow-up of 1057 (915-1321) days.

### Acute irAEs

Of all 318 patients, 226 (71.1%) experienced acute irAEs and 53 (17.0%) experienced delayed toxic effects (Figure and Table 1). Most acute irAEs were grade 2 or above (144 patients [63.7%]) with 114 patients (50.4%) requiring steroids. The most common acute irAEs included dermatitis or pruritus (91 patients [28.6%]), thyroiditis or hypothyroid (49 patients [15.4%]), arthritis or arthralgias (46 patients [14.5%]), and colitis or diarrhea (33 patients [10.4%]).

**Table 1.** Acute and Delayed irAEs Arising in 318 Patients Treated With Anti–Programmed Cell Death-1 Therapy

Acute irAE incidence	Patients, No. (%)	
	Acute toxic effects	Delayed toxic effects
irAE count, No.	226	53
Grade ≥2	144 (63.7)	38 (71.7)
Grades 3-5	44 (19.5)	14 (26.4)
Toxicity requiring steroids	114 (50.4)	29 (54.7)
Grade 2	102 (45.1)	28 (52.8)
Grades 3-5	39 (17.3)	12 (22.6)

Abbreviation: irAEs, immune-related adverse events.

### Chronic irAEs

We assessed how often acute irAEs, including delayed irAEs, became chronic. Adrenal insufficiency (8 of 10 patients [80.0%]), hypophysitis (8 of 8 patients [100.0%]), thyroiditis/hypothyroid (48 of 56 patients [85.7%]), neuropathy (6 of 7 patients [85.7%]), and nephritis (4 of 5 patients [80%]) appeared particularly likely to evolve into chronic irAEs, similar to our prior report (Table 2). Colitis or diarrhea (5 of 40 patients [12.5%]), dermatitis or pruritus (21 of 95 patients [22.1%]), hepatitis (4 of 22 patients [18.2%]), and pneumonitis (8 of 18 patients [44.4%]) had lower rates of chronicity. Of note, 12 patients (3.8%) had fatigue listed as a chronic toxicity, however, given the difficulty with attributing such a toxicity, we did not include this in our statistical analysis.

**Table 2.** Acute and Delayed irAEs Persisting to Chronic irAEs

Toxicity types	No. of patients (% of total patients)	Developed delayed irAE after ICI therapy (% of total patients)	Persisted to a chronic irAE (% of patients with acute toxicity) with >1.5 y follow-up
Adrenal insufficiency	6 (1.9)	4 (1.3)	8 (80.0)
Arthritis/arthralgias	46 (14.5)	7 (2.2)	26 (49.1)
Bullous pemphigoid	2 (0.6)	1 (0.3)	2 (66.7)
Colitis/diarrhea	33 (10.4)	7 (2.2)	5 (12.5)
Cough	2 (0.6)	0	2 (100.0)
Dermatitis/pruritus	91 (28.6)	4 (1.3)	21 (22.1)
Diabetic ketoacidosis	1 (0.3)	0	0
Xerostomia^a^	15 (4.7)	3 (0.9)	10 (55.6)
Esophagitis	1 (0.3)	0	1 (100.0)
Gastritis/enteritis	5 (1.6)	1 (0.3)	2 (33.3)
Hematologic toxic effects^b^	1 (0.3)	2 (0.6)	0
Hepatitis	21 (6.6)	1 (0.3)	4 (18.2)
Hypophysitis	6 (1.9)	2 (0.6)	8 (100.0)
Hypogonadism	1 (0.3)	0	0
Mucositis	3 (0.9)	2 (0.6)	2 (40.0)
Myalgias	6 (1.9)	0	0
Myocarditis	2 (0.6)	0	0
Myositis	2 (0.6)	0	0
Nausea	3 (0.9)	0	0
Nephritis/nephrotic syndrome	2 (0.6)	3 (0.9)	4 (80.0)
Neuropathy	7 (2.2)	0	6 (85.7)
Ocular toxic effects^c^	7 (2.2)	0	4 (57.1)
Other neurotoxicity^d^	5 (1.6)	3 (0.9)	6 (75.0)
Pancreatitis or pancreatic insufficiency	1 (0.3)	1 (0.3)	1 (50.0)
Pneumonitis	14 (4.4)	4 (1.3)	8 (44.4)
Psoriasis	4 (1.3)	0	1 (25.0)
Raynaud	1 (0.3)	0	1 (100.0)
Syncope	1 (0.3)	0	0
Thyroiditis/hypothyroid	49 (15.4)	7 (2.2)	48 (85.7)
Vitiligo	4 (1.3)	4 (1.3)	6 (75.0)
Other^e^	8 (2.5)	1 (0.3)	2 (22.2)

Abbreviations: ICI, immune checkpoint inhibitor; irAEs, immune-related adverse events.

^a^ Acute xerostomia (12 patients), sicca (1 patient), Sjogrens (2 patients), delayed xerostomia (3 patients), chronic xerostomia (10 patients).

^b^ Acute pancytopenia (1 patient), delayed neutropenia (2 patients), no chronic.

^c^ Acute retinal vasculitis (1 patient), conjunctivitis (3 patients), uveitis (1 patient), vitreitis (1 patient), nonischemic optic neuritis (1 patient), chronic retinal vasculitis (1 patient), nonischemic optic neuritis (1 patient), uveitis (1 patient), conjunctivitis (1 patient).

^d^ Acute tremor (1 patient), Guillain-Barre syndrome (1 patient), headache (3 patients), delayed autonomic dysfunction (2 patients), Frey syndrome (1 patient), chronic autonomic dysfunction (2 patients), tremor (1 patient), headache (1 patient), Frey syndrome (1 patient), Guillain-Barre syndrome (1 patient).

^e^ Acute sinusitis (2 patients), flu-like symptoms (2 patients), constipation (1 patient), hyperglycemia (1 patient), dyspnea (1 patient), heartburn (1 patient), delayed constipation (1 patient), chronic sinusitis (2 patients).

Among all 318 patients, 147 patients (46.2%; 95% CI, 0.41%-0.52%) developed chronic toxic effects, 74 (50.3%) of those patients (or 23.3% of the full cohort) had at least grade 2, and 6 (4.1%) of those patients (or 1.9% of the full cohort) were grade 3 to 5 (Figure and Table 3). Of all patients with chronic toxic effects, 56 (38.1%) required corticosteroids, and 29 patients (19.7%) required steroids for greater than grade 2 or more toxic effects and 2 (1.4%) patients grade 3 or more toxic effects. Additionally, 100 patients (68.0%) experienced symptomatic chronic toxic effects. The median (IQR) duration of treatment was 315 (68-352) days (mean [SD], 265 [116.7] days) for patients with chronic irAEs and 315 (169-351) days (mean [SD], 260 [117.9] days) for patients without chronic irAEs.

**Table 3.** Incidence of Chronic irAEs

Incidence	Patients with chronic toxic effects, No. (%)
Any chronic irAE, No.	147
Grade ≥2	74 (50.3)
Grades 3-5	6 (4.1)
Toxicity requiring steroids	56 (38.1)
Toxicity requiring steroids (grade 2)	29 (19.7)
Toxicity requiring steroids (grades 3-5)	2 (1.4)
Symptomatic	100 (68.0)
Symptomatic endocrinopathy	20 (13.6)
Resolved	52 (35.4)
Persisted	95 (64.6)

Abbreviation: irAEs, immune-related adverse events.

Among 75 patients with acute endocrine irAEs, 64 (85.3%) became chronic, 11 of which also had nonendocrine irAEs. Among chronic endocrine irAEs, 20 (31.3%) were symptomatic, 55 (85.9%) were grade 2 or more, and 1 (1.6%) was grade 3 (hypophysitis). Additionally, 94 of 244 (38.5%) patients with acute nonendocrine irAEs developed chronic irAEs, of which 86 (91.5%) were symptomatic, 26 (27.7%) were grade 2 or more, and 6 (6.4%) were grade 3 to 5.

### Outcome of Chronic irAEs

With prolonged follow-up (median [IQR], 1057 [915-1321] days), 54 of 147 patients (36.7%) experienced resolution of chronic irAEs at last follow-up, while 93 of 147 patients with chronic toxic effects (63.3% of the cohort [95% CI, 0.55-0.71] or 29.2% of original cohort [95% CI, 0.25-0.34]) were ongoing (Figure and Table 4). The median (IQR) time to resolution of chronic irAEs was 19.7 (14.4-31.5) months from the time of anti–PD-1 start and 11.2 (8.1-20.7) months from the time of anti–PD-1 cessation. Results were similar when considering only patients with more than 2 years of follow-up; 136 (42.8%) patients developed chronic irAEs, 50 (36.8%) of which resolved by last follow-up (eTable 2 in Supplement 1).

**Table 4.** Chronic irAEs Persisting with Long-Term Follow-Up

Toxicity types^a^	No. of patients (% of total patients)	Ongoing at last follow up (>1.5 y) (% of patients with chronic toxicity)
Adrenal insufficiency	8 (2.5)	8 (100.0)
Arthritis/arthralgias	26 (8.2)	18 (69.2)
Colitis/diarrhea	5 (1.6)	3 (60.0)
Dermatitis/pruritus	21 (6.6)	9 (42.9)
Xerostomia	10 (3.1)	6 (60.0)
Hepatitis	4 (1.3)	0
Hypophysitis	8 (2.5)	8 (100.0)
Nephritis/nephrotic syndrome	4 (1.3)	2 (50.0)
Neuropathy	6 (1.9)	4 (66.7)
Ocular toxic effects^b^	4 (1.3)	2 (50.0)
Other neurotoxicity^c^	6 (1.9)	3 (50.0)
Pneumonitis	8 (2.5)	3 (37.5)
Thyroiditis/hypothyroid	48 (15.1)	38 (79.2)
Vitiligo	6 (1.9)	4 (66.7)

Abbreviation: irAEs, immune-related adverse events.

^a^ Observed at least 1% frequency.

^b^ Chronic retinal vasculitis (1 patient), nonischemic optic neuritis (1 patient), uveitis (1 patient), conjunctivitis (1 patient), ongoing at last follow-up retinal vasculitis (1 patient), nonischemic optic neuritis (1 patient).

^c^ Chronic autonomic dysfunction (2 patients), tremor (1 patient), headache (1 patient), Frey syndrome (1 patient), ongoing at last follow-up autonomic dysfunction (1 patient), headache (1 patient), Guillain-Barre syndrome (1 patient). Several patients experienced multiple chronic irAEs.

Among the 93 patients with persistent toxic effects present at last follow-up, 55 (59.1% or 17.3% of original cohort) were grade 2 or above and 41 (44.1% or 12.9% of original cohort) remained symptomatic (eTable 4 in Supplement 1). Of these, 54 patients (58.1%) experienced persistent endocrinopathies, 48 (51.6%) experienced nonendocrinopathies, and 9 (9.7%) experienced both. Overall 54 of 64 patients with chronic endocrine irAEs (84.4%) experienced persistent irAEs at the time of last follow-up; 48 of 94 patients with chronic nonendocrine irAEs (51.1%) experienced persistent irAEs, of which 33 (68.8%) were symptomatic, 16 (33.3%) were grade 2 or above, and 2 (4.2%) were grade 3 to 5 (grade 3 colitis and CKD). The median (IQR) time to chronic nonendocrine irAE resolution was 19.5 (14.1-29.9) months from the time of anti–PD-1 start and 9.0 (7.0-20.1) months from the time of anti–PD-1 cessation. In the 10 patients with resolution, the median (IQR) time to chronic endocrine irAE resolution was 38.9 (25.2-52.7) months from the time of anti–PD-1 start and 28.8 (13.4-41.7) months from the time of anti–PD-1 cessation. Among the 54 patients with persistent endocrinopathies, 16 (29.6%) were receiving steroids and 27 (50.0%) were receiving other management (eg, levothyroxine, pancreatic enzymes, etc). Of 48 patients with persistent nonendocrinopathies, 7 (14.6%) were receiving steroids and 17 (35.4%) were receiving other treatment (eg, gabapentin, methotrexate, hydroxychloroquine). Notably, hypothyroid (38 [40.9%]), adrenal insufficiency (8 [8.6%]), arthritis (18 [19.4%]), dermatitis or pruritus (9 [0.7%]), and hypophysitis (8 [8.6%]) represented the majority of persistent irAEs. Of 6 patients with chronic grade 3 to 5 irAEs, 3 (50.0%) showed improvement (grade 3 esophagitis, bullous pemphigoid, and arthritis), 1 (16.7%) experienced resolution (grade 4 nephritis), and 2 (33.3%) had ongoing grade 3 irAEs at last follow-up (grade 3 hypophysitis or colitis and chronic kidney disease).

Compared with 77 patients without chronic irAEs (45.0%), 48 patients with chronic irAE (32.7%) developed disease recurrence, 18 (12.2%) had regional recurrence, and 30 (20.4%) had metastatic recurrence (eTable 5 in Supplement 1). Of 37 patients with chronic irAEs who received additional immunotherapy, 25 patients (67.6%) experienced no effect on chronic irAEs, 12 patients (32.4%) experienced a flare in their chronic irAEs, and 20 patients (54.1%) experienced distinct irAEs (eTable 6 in Supplement 1). In an exploratory analysis, patients with chronic irAEs demonstrated longer median RFS and overall survival compared with those without chronic irAEs (eTable 7 and eFigure in Supplement 1).

## Discussion

In this cohort study of 318 patients treated with adjuvant anti–PD-1, chronic irAEs were common and often persisted long-term, although many chronic, nonendocrine irAEs eventually resolved. With at least 18 months of follow-up, 35.4% of patients experienced resolution of their chronic irAEs (including nearly half of nonendocrine irAEs), increased from our previous study showing that 14.4% of chronic toxic effects resolved with at least 6 months follow-up. While endocrine toxic effects (adrenal insufficiency, hypophysitis, and thyroiditis or hypothyroid) were more likely to become chronic and persist at last follow-up than nonendocrine toxic effects, other more symptomatic irAEs persisted at low rates individually, including cutaneous, rheumatologic, oral, and ocular events.

We and others have previously speculated whether chronic irAEs represent smoldering autoimmunity vs a burnout phenotype, where all the relevant cells have been destroyed or damaged, leading to continued symptoms.^9^ The resolution of some chronic irAEs, as well as the flares occurring with retreatment (32.4%) suggest some patients have ongoing inflammation. The persistent nature of irAEs, particularly endocrinopathies, suggests that permanent damage may occur in some patients. For this population, rechallenge with a lower risk of irAEs may be possible (although some still flare).^10^ Further research should aim to identify patients that are predisposed to persistent toxic effects.

### Limitations

This study had limitations. Review of toxic effects and adverse events using clinical data and notes may lack the fidelity of formal clinical trial adverse event reporting. Documentation may not mention every toxicity in every note, thus lowering the fidelity of the resolution date for individual patients. While most patients overlap with our prior study, the departure of 1 investigator from their prior institution resulted in slightly distinct patient populations from our previous study. RFS analysis between patients with and without chronic irAEs is highly likely to suffer from time-dependent bias.

## Conclusions

Insights into the long-term impact of adjuvant anti–PD-1 therapy are crucial to optimize patient outcomes as these agents are used across different tumor types. The high prevalence of chronic irAEs suggests the importance of considering the risk-benefit ratio when initiating adjuvant therapy and the need for prolonged monitoring and proactive management of irAEs.

## Notes

Supplement 1. **eTable 1.** Acute Immune-Related Adverse Events (irAEs) Arising During Anti–Programmed Cell Death 1 Therapy **eTable 2.** Incidence of Chronic Immune-Related Adverse Events (irAEs) **eTable 3. **Chronic Immune-Related Adverse Events (irAEs) Persisting at Last Follow-Up **eTable 4.** Chronic Immune-Related Adverse Events (irAEs) Features at Time of Last Follow-Up **eTable 5.** Chronic Immune-Related Adverse Events (irAEs), Disease Recurrence, and Additional Immunotherapy Effects **eTable 6.** Effect of Additional Immunotherapy on Chronic irAEs **eTable 7.** Chronic Immune-Related Adverse Events (irAEs) and Immunotherapy Outcomes **eFigure.** Kaplan-Meier Estimates of Overall Survival (OS) and Relapse-Free Survival (RFS)

Supplement 2.
Data Sharing Statement
