# Supplementary material for: Extended Follow-Up of Chronic Immune-Related Adverse Events Following Adjuvant Anti–PD-1 Therapy for High-Risk Resected Melanoma
Source: JAMA Netw Open. 2023 Aug 3;6(8):e2327145. doi: 10.1001/jamanetworkopen.2023.27145 (PMC10401300; doi:10.1001/jamanetworkopen.2023.27145)
Supplement: Supplement 1. — eTable 1. Acute Immune-Related Adverse Events (irAEs) Arising During Anti–Programmed Cell Death 1 Therapy eTable 2. Incidence of Chronic Immune-Related Adverse Events (irAEs) eTable 3. Chronic Immune-Related Adverse Events (irAEs) Persisting at Last Follow-Up eTable 4. Chronic Immune-Related Adverse Events (irAEs) Features at Time of Last Follow-Up eTable 5. Chronic Immune-Related Adverse Events (irAEs), Disease Recurrence, and Additional Immunotherapy Effects eTable 6. Effect of Additional Immunotherapy on Chronic irAEs eTable 7. Chronic Immune-Related Adverse Events (irAEs) and Immunotherapy Outcomes eFigure. Kaplan-Meier Estimates of Overall Survival (OS) and Relapse-Free Survival (RFS) [file jamanetwopen-e2327145-s001.pdf]

Supplemental Online Content

Goodman RS, Lawless A, Woodford R, et al. Extended follow-up of chronic immune-related adverse events following adjuvant anti–PD-1 therapy for high-risk resected melanoma. *JAMA Netw Open*. 2023;6(8):e2327145.
doi:10.1001/jamanetworkopen.2023.27145

eTable 1. Acute Immune-Related Adverse Events (irAEs) Arising During Anti–Programmed Cell Death 1 Therapy
eTable 2. Incidence of Chronic Immune-Related Adverse Events (irAEs)
eTable 3. Chronic Immune-Related Adverse Events (irAEs) Persisting at Last Follow-Up
eTable 4. Chronic Immune-Related Adverse Events (irAEs) Features at Time of Last Follow Up
eTable 5. Chronic Immune-Related Adverse Events (irAEs), Disease Recurrence, and Additional Immunotherapy Effects
eTable 6. Effect of Additional Immunotherapy on Chronic irAEs
eTable 7. Chronic Immune-Related Adverse Events (irAEs) and Immunotherapy Outcomes
eFigure. Kaplan-Meier Estimates of Overall Survival (OS) and Relapse-Free Survival (RFS)

This supplemental material has been provided by the authors to give readers additional information about their work.

© 2023 Goodman RS et al. *JAMA Network Open.*

eTable 1. Acute Immune-Related Adverse Events (irAEs) Arising During Anti–Programmed Cell Death 1 Therapy

**Table 1: Patient and disease characteristics (> 1.5 year follow up)**	**Number of patients (%)**
**General demographics**	
Gender (male)	190 (59.7)
Age (years; median, range)	61, 21-88
**Primary Site**	
Cutaneous	270 (84.9)
Acral	19 (6.0)
Mucosal	3 (0.9)
Unknown primary	26 (8.2)
**Mutation Status**	
BRAF	70 (22.0)
NRAS	61 (19.2)
WT	171 (53.8)
Unknown	12 (3.8)
**Stage at presentation**	
II	6 (1.9)
III (n=2 (unknown primary), n=1 (mucosal))	3 (0.9)
IIIA	30 (9.4)
IIIB	113 (35.5)
IIIC	124 (39.0)
IIID	9 (2.8)
IV	33 (10.4)
**Treatment**	
Pembrolizumab	63 (19.8)
Nivolumab	255 (80.2)
**Duration on therapy (days; median)**	315
**Reasons for stopping therapy**	
Progression	56 (17.6)
Toxicity	76 (23.9)
Completed therapy	170 (53.5)
Other[a]	16 (5.0)
**Disease recurrence**	
No recurrence	193 (60.7)

© 2023 Goodman RS et al. *JAMA Network Open.*

Regional recurrence	56 (17.6)
Metastatic recurrence	69 (21.7)
**Follow-up**	
Median duration of follow-up (days)	1353
Median duration of follow-up after treatment cessation (days)	1057
Alive at last follow-up	284 (89.3)

[a]Other includes reasons related to the COVID-19 pandemic, patient preferences, funding, comorbidities

© 2023 Goodman RS et al. *JAMA Network Open.*

eTable 2. Incidence of Chronic Immune-Related Adverse Events (irAEs)

	**Number of patients (% of those with chronic irAE)**	
	>1.5 year follow-up	>2 year follow-up
Any chronic irAE	147	136
Grade ≥ 2	74 (50.3)	67 (49.3)
Grades 3-5	6 (4.1)	5 (3.7)
Toxicity requiring steroids	56 (38.1)	52 (38.2)
Toxicity requiring steroids (grade 2)	29 (19.7)	26 (19.1)
Toxicity requiring steroids (grades 3-5)	2 (1.4)	2 (1.5)
Symptomatic	100 (68.0)	92 (67.6)
Symptomatic endocrinopathy (adrenal insufficiency, hypophysitis, thyroiditis, pancreatic insufficiency)	20 (13.6)	17 (12.5)
Resolved	54 (36.7)	50 (36.8)
Persisted	93 (63.3)	86 (63.2)

© 2023 Goodman RS et al. *JAMA Network Open.*

eTable 3. Chronic Immune-Related Adverse Events (irAEs) Persisting at Last Follow-Up

Toxicity types	Number of patients (% of total patients)	Ongoing at last follow up (% of patient with chronic irAE) >1.5 yr f/u)	Ongoing at last follow up (% of patient with chronic irAE) (>2 yr f/u)
Adrenal insufficiency	8 (2.5)	8 (100.0)	7 (100.0)
Arthritis/arthralgias	26 (8.2)	18 (69.2)	17 (70.8)
Bullous pemphigoid	2 (0.6)	1 (50.0)	1 (50.0)
Colitis/diarrhea	5 (1.6)	3 (60.0)	2 (50.0)
Cough	2 (0.6)	0 (0.0)	0 (0.0)
Dermatitis/pruritis	21 (6.6)	9 (42.9)	8 (42.1)
Xerostomia[a]	10 (3.1)	6 (60.0)	5 (55.6)
Esophagitis	1 (0.3)	1 (100.0)	1 (100.0)
Gastritis/enteritis	2 (0.6)	1 (50.0)	1 (50.0)
Hepatitis	4 (1.3)	0 (0.0)	0 (0.0)
Hypophysitis	8 (2.5)	8 (100.0)	7 (100.0)
Mucositis	2 (0.6)	2 (100.0)	1 (100.0)
Nephritis/nephrotic syndrome	4 (1.3)	2 (50.0)	2 (50.0)
Neuropathy	6 (1.9)	4 (66.7)	4 (66.7)
Ocular toxicities[b]	4 (1.3)	2 (50.0)	2 (50.0)
Other neurotoxicity[c]	6 (1.9)	3 (50.0)	2 (40.0)
Pancreatitis or pancreatic insufficiency	1 (0.3)	1 (100.0)	1 (100.0)
Pneumonitis	8 (2.5)	3 (37.5)	3 (37.5)
Psoriasis	1 (0.3)	0 (0.0)	0 (0.0)
Raynaud's	1 (0.3)	0 (0.0)	0 (0.0)
Thyroiditis/hypothyroid	48 (15.1)	38 (79.2)	35 (77.8)
Vitiligo	6 (1.9)	4 (66.7)	4 (66.7)
Sinusitis	2 (0.6)	0 (0.0)	0 (0.0)

Several patients experienced multiple chronic irAEs.

[a] (ongoing at last follow up (>1.5 yrs) xerostomia (5), Sjogren's (1))

[b]Chronic - retinal vasculitis (1), non-ischemic optic neuritis (1), uveitis (1), conjunctivitis (1), ongoing at last follow-up - retinal vasculitis (1), non-icshemic optic neuritis (1)

[c]Chronic - autonomic dysfunction (2), tremor (1), Headache (1), Frey's syndrome (1), Guillain-Barre Syndrome (1), ongoing at last follow up - autonomic dysfunction (1), headache (1), Guillain-Barre Syndrome (1)

© 2023 Goodman RS et al. *JAMA Network Open.*

eTable 4. Chronic Immune-related Adverse Events (irAEs) Features at time of Last Follow Up

**Chronic irAE (% of those with chronic toxicities present at last follow up, n=93)**		
	>1.5 year follow up	>2 year follow up
Grade ≥ 2	55 (59.1)	50 (58.1)
Grade 3-5	2 (2.2)	1 (1.2)
Endocrine irAE	54 (58.1)	49 (57.0)
Non-endocrine irAE	48 (51.6)	44 (51.2)
On steroids at last follow up	24 (25.8)	22 (25.6)
On replacement steroids at last follow up	16 (17.2)	14 (16.3)
On other management at last follow up	42 (45.2)	39 (45.3)
Symptomatic	41 (44.1)	39 (45.3)

© 2023 Goodman RS et al. *JAMA Network Open.*

eTable 5. Chronic Immune-related adverse events (irAEs), Disease Recurrence, and Additional Immunotherapy Effects

**Chronic irAEs (% patients with chronic irAEs)**	>1.5 year follow-up	>2 year follow-up
Recurrence	48 (32.7)	41 (30.1)
Regional	18 (12.2)	15 (11.0)
Metastatic	30 (20.4)	26 (19.1)
Required additional immunotherapy	37 (25.2)	33 (23.5)
**Effect of additional immunotherapy on chronic toxicity (% patients with chronic toxicities requiring additional immunotherapy)[a]**		
No effect	25 (67.6)	24 (72.7)
Chronic toxicity flared	12 (32.4)	9 (27.3)
Distinct toxicity	20 (54.1)	18 (54.5)

[a]one patient experienced both a chronic toxicity flare and distinct toxicity

© 2023 Goodman RS et al. *JAMA Network Open.*

eTable 6. Effect of Additional Immunotherapy on Chronic irAEs

Chronic irAEs that flared with additional immunotherapy	Count
Adrenal insufficiency	1
Arthritis/arthralgias[a]	1
Bullous pemphigoid	1
Colitis	2
Dermatitis	4
Mucositis	1
Thyroiditis	1
Vitiligo	1

Chronic irAEs that did not flare with additional immunotherapy	Count
Arthritis/arthralgias	3
Xerostomia	1
Hepatitis	2
Hypophysitis	1
Neuropathy	2
Ocular toxicities	2
Thyroiditis	14

[a]The patient experienced a flare of arthralgias.

© 2023 Goodman RS et al. *JAMA Network Open.*

eTable 7. Chronic Immune-related Adverse Events (irAEs) and Immunotherapy Outcomes

	Total patients		Patient with Chronic IrAEs	
	**>1.5 yr follow up**	**>2 yr follow up**	**>1.5 yr follow up**	**>2 yr follow up**
**RFS[a, b], days (median (IQR[a]))**	1242 (863)	1269 (807)	1290 (756)	1333 (500)
**OS[a, b], days (mean, median)**	1354 (340)	1386 (343)	1390 (331)	1411 (300)

[a]Abbreviations: RFS, recurrence free survival; IQR, interquartile range; OS, overall survival
[b]Start time as date of anti-PD-1 initiation

© 2023 Goodman RS et al. *JAMA Network Open.*

eFigure. Kaplan-Meier Estimates of Overall Survival (OS) and Relapse-Free Survival (RFS)

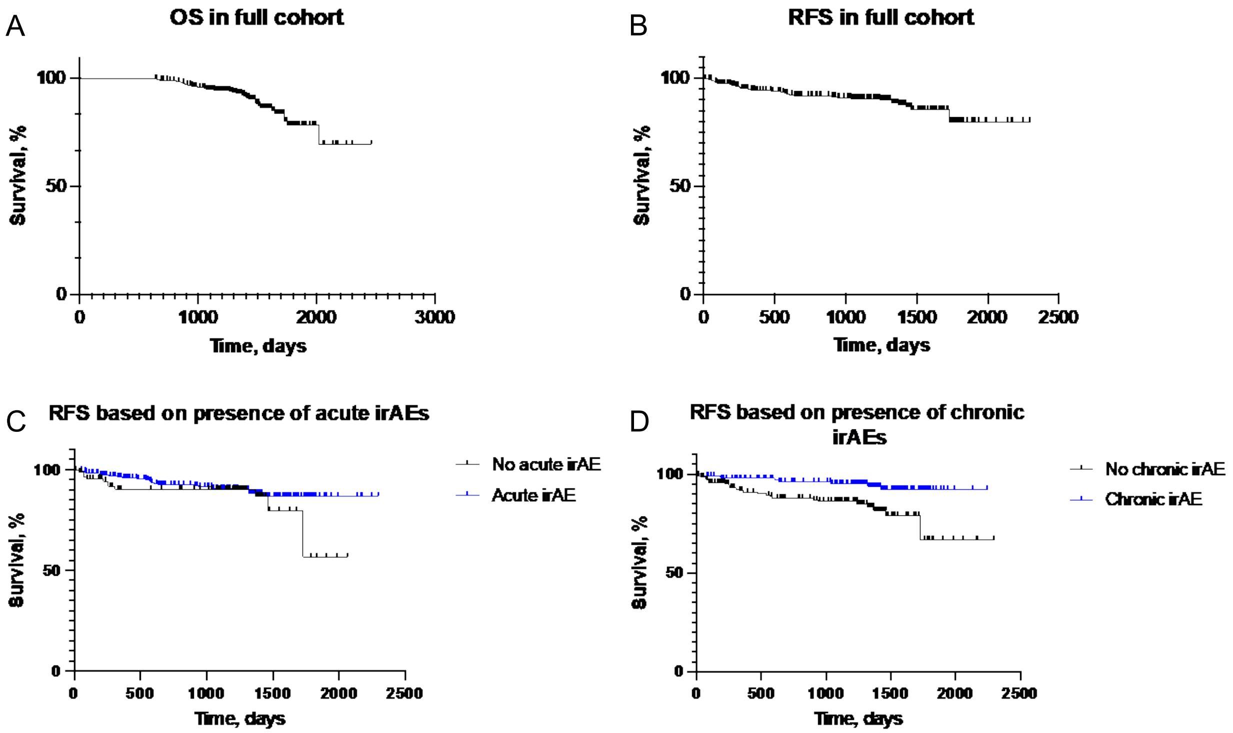

© 2023 Goodman RS et al. *JAMA Network Open.*
