# Supplementary material for: Extended Follow-Up of Chronic Immune-Related Adverse Events Following Adjuvant Anti–PD-1 Therapy for High-Risk Resected Melanoma
Source: JAMA Netw Open. 2023 Aug 3;6(8):e2327145. doi: 10.1001/jamanetworkopen.2023.27145 (PMC10401300; doi:10.1001/jamanetworkopen.2023.27145)
Supplement: Supplement 2. — Data Sharing Statement [file jamanetwopen-e2327145-s002.pdf]

Data Sharing Statement

Data
Data available: No

Additional Information
Explanation for why data not available: Detailed clinical data, difficult to completely anonymize
